# Perceptions of the COVID-19 Vaccine and Other Adult Vaccinations in Malawi: A Qualitative Assessment

**DOI:** 10.9745/GHSP-D-23-00146

**Published:** 2024-02-20

**Authors:** Natalie Tibbels, Rominie Kaseghe, Alvin Blessings Chisambi, Vitima Ndovi, Alfred Mang’ando, Maria Elena Figueroa

**Affiliations:** a Johns Hopkins Bloomberg School of Public Health, Baltimore, MD, USA.; b Johns Hopkins Center for Communication Programs-Malawi, Lilongwe, Malawi.

## Abstract

In Malawi, various brands of the COVID-19 vaccine have been offered to the population, but factors including fear of side effects or other risks, uncertainty about benefits, and misinformation created hesitancy toward them. In early 2022, 4% of Malawians were fully vaccinated for COVID-19. Despite multiple promotion efforts, by August 2022, COVID-19 vaccination nationwide was around 15%. To increase COVID-19 vaccination uptake, the research team collected qualitative data in 4 districts with vaccine coverage levels ranging from 1% to 11%. This data collection happened during a cholera outbreak that began in March 2022 and the vaccination efforts to address it. Study participants included male and female members of the general population, social workers, people with comorbidities, health workers, and community leaders (224 participants total, 47% female). In focus group discussions (n=27) and in-depth interviews (n=17), participants compared COVID-19 vaccines with other adult vaccines, such as cholera and tetanus toxoid. A thematic analysis identified themes related to 3 research questions on COVID-19 vaccine concerns, confidence, and delivery affecting uptake. Differences in promotion, delivery (oral versus injection), COVID-19 vaccine card structure, the various brands and boosters, and vaccines being described as required or optional all played a role in distinguishing COVID-19 vaccines from other vaccines and creating suspicion or indifference. Barriers to vaccination in general, such as rumors or knowledge gaps, were amplified by how novel the COVID-19 vaccines were perceived to be and the changing guidance provided over time. By April 2023, more targeted campaign efforts helped increase vaccination rates to 28%. The findings contribute information about how individuals conceptualize and make decisions about adult vaccination, which can, in turn, inform strategies to integrate COVID-19 promotion and delivery with other disease responses in Malawi as well as routine health services in similar settings.

## Abstract (precis)

Low perceived risk of COVID-19 and fear of the COVID-19 vaccines suggest the utility of integrating COVID-19 vaccine delivery into other health services and using trusted community-based vaccinators who emphasize the public good and avoid framing COVID-19 vaccines as different from other vaccines.

## Teaser

**Key Findings:**

- Though Malawi faced a “twindemic” of COVID-19 and cholera, participants in this qualitative study tended to fear cholera and other health issues more than COVID-19, leading to low uptake of COVID-19 vaccines.
- Convenient access to COVID-19 vaccines in one’s home or while accessing other health services, encouragement from health workers to vaccinate that was perceived to be sincere, and an understanding of the benefits of the vaccines all contributed to vaccine confidence.
- Specific elements of COVID-19 vaccine delivery strategies—such as having a separate vaccination card and an emphasis on the individual’s choice to get vaccinated—set COVID-19 vaccines apart from other adult vaccinations and contributed to low vaccine uptake.

**Key Implication:**

- Public health practitioners should bundle COVID-19 vaccination strategies with other, more trusted vaccinations and strengthen messaging about the benefits of vaccination for the public good.

Plain language article summary available.

## INTRODUCTION

Health threats do not occur in clean, siloed, or sequential experiences for individuals and communities. “Twindemics”—when 2 epidemics converge on the same location at the same time—compound negative health outcomes, as resources are divided, communication is muddled, or people avoid routine health services.^1^^,^^2^ Malawi faced a twindemic—battling a cholera outbreak beginning in March 2022 while COVID-19 was still a global threat and as health and economic systems already strained by the COVID-19 pandemic were reaching a breaking point.^3^ In April 2023, as the cholera outbreak was ongoing, Malawi registered a total of 88,620 confirmed COVID-19 cases, including 2,686 deaths.^4^

Since the 1970s and increasingly since 1998, Malawi has had intermittent cholera cases, particularly during the wet season and in flood-prone areas, and has used both emergency and mass vaccination strategies for oral cholera vaccine (OCV).^5^ On March 3, 2022, the country declared a cholera outbreak after a confirmed case at Machinga District Hospital. By April 2023, all districts in the country were affected, and by August, the country had experienced almost 59,000 cumulative cholera cases and 1,768 deaths.^3^ The outbreak in Malawi tended to affect younger people, with more than half of cases in individuals aged 10–29 years but more deaths in individuals aged 60 years and older.^3^ The cholera outbreak had a case fatality rate of 3.0%, with Lilongwe, the capital, and Blantyre, the second largest city, recording the highest number of deaths.

In light of how widespread and severe the cholera outbreak became, the government recognized the urgent need to improve access to safe water, sanitation, and hygiene to ensure interdistrict and cross-border coordination and collaboration and to invest in risk communication and community engagement (RCCE) as part of its response. Malawi also received OCV doses from Gavi, the Vaccine Alliance and began a campaign to vaccinate more than 1.9 million people beginning in May 2022; vaccination campaigns offered OCV to adults and children aged 1 year and older living in high-risk areas.^3^ In April 2023, an additional 1.4 million doses were approved by the International Coordinating Group with support from Gavi after the impact of Tropical Cyclone Freddy that hit 15 districts in Malawi in February and March 2023.^6^

COVID-19 vaccination officially launched in March 2021, and the total number of fully vaccinated people went from 4% in March 2022 to 15% in August 2022 to almost 28% of the eligible population by April 2023, according to bulletins from the Public Health Institute of Malawi.^7^ In February 2023, the government of Malawi launched the End Cholera Campaign to deliver a package of integrated health interventions. To this end, the government began to integrate cholera and COVID-19 responses with combined district-led campaigns, where COVID-19 vaccines were offered at the same time as oral rehydration salts, chlorine tablets, and education on cholera prevention.

Limited research has been conducted in Southern Africa on how people conceptualize COVID-19 vaccination relative to other types of vaccines, particularly in an emergency setting. Current evidence suggests a link between intention to receive the COVID-19 vaccine and intentions or experience with other routine vaccinations or compliance with other disease prevention measures.^8^^,^^9^ The World Health Organization’s Behavioral and Social Drivers Framework lists “thinking and feeling” constructs as key drivers of vaccine intention, specifically listing perceived disease risk and vaccine confidence (including perceived benefits, safety, and trust).^10^

Limited research has been conducted in Southern Africa on how people conceptualize COVID-19 vaccination relative to other types of vaccines.

To explore COVID-19 vaccine perceptions, the Breakthrough ACTION COVID-19 Vaccine project in Malawi worked with the Malawi Ministry of Health (MOH) to collect qualitative data to explore vaccine confidence and access and particularly investigate people’s views of COVID-19 vaccines compared to other vaccines that they regularly received and the modes in which they were provided. For this article, we conducted a secondary analysis of these data to explore in more detail what participants in focus group discussions (FGDs) and in-depth interviews (IDIs) reflected upon regarding the COVID-19 vaccine and other vaccines available in Malawi. The results aim to add to the lessons learned about challenges that the COVID-19 vaccine faced and how this and future emergency vaccines could be introduced and integrated with existing vaccination schedules.

## METHODS

The Breakthrough ACTION COVID-19 Vaccine project in Malawi was a 2-year project (April 2021–June 2023) funded by the U.S. Agency for International Development led by the Johns Hopkins University Center for Communication Programs in partnership with Save the Children Malawi to implement activities at the national and community levels. The Breakthrough ACTION team worked with Health Education Services, a department within the MOH, to develop a variety of communication products for mass media and social media and implemented community engagement activities to create demand for COVID-19 vaccines.

### Qualitative Assessment

As part of the larger project and in consultation with the MOH, the Breakthrough ACTION team used qualitative methods to explore individual COVID-19 vaccine perceptions, the social context, and perceived norms around COVID-19 vaccination. The assessment comprised IDIs with health workers and community gatekeepers (e.g., chiefs, village heads, or their representatives) and FGDs with unvaccinated and vaccinated men and women, social workers, and individuals with comorbidities, such as diabetes or hypertension. Social workers were included because they played an important role in connecting community members to social services, particularly during the pandemic. IDI guides explored the perceived relevance of COVID-19 among other health concerns in communities, the process of vaccine promotion and delivery, and participants’ perceptions of how accessible the vaccines were. FGD guides explored a variety of factors that may influence uptake of the COVID-19 vaccine and began with a sorting exercise where participants collaboratively listed health issues that people in the community were concerned about and then ranked the issues in order of most concern. If not mentioned, COVID-19 was added to the list by the FGD facilitator to understand factors that influence risk perception. Both IDI and FGD guides were revised and validated by the MOH during a 1-day collaborative review workshop. The MOH also delegated officials from its units (the Malawi Public Health Institute, the Health Education Unit, and the Essential Programme on Immunization) to facilitate sessions on technical topics during the training of data collectors. Officials also connected the data collection team with district and community leaders to ensure appropriate community entry.

### Setting and Sample

Data were collected in August 2022 in 4 districts. The districts were selected for their regional spread, availability of partners to apply the findings, and COVID-19 vaccination coverage at the time: Ntchisi (Central Region, top performing on COVID-19 vaccination, 11.4% coverage), Thyolo (Southern Region, lowest performing with 1.2% COVID-19 vaccine coverage), Machinga (Eastern Region, middle performance with 6.5% coverage, and the origin of the 2022–2023 cholera outbreak), and Karonga (Northern Region, middle performance at 5.2%). These districts, although not statistically representative of the country, reflected the diverse scenarios regarding COVID-19 vaccination in Malawi and where RCCE partners were in place to act on the findings. Across the 4 districts, the data collection team aimed to conduct 12 FGDs with vaccinated men and women, 10 FGDs with unvaccinated men and women, 2 FGDs with social workers, and 2 with people with comorbidities, along with 8 IDIs with health workers and 8 IDIs with community gatekeepers.

The qualitative study used purposive sampling, recruiting potential participants with the help of district health officers and social welfare officers, community health volunteers, and health surveillance assistants (HSAs). A standard recruitment script explained the study’s purpose and the procedures that potential participants could expect. For FGDs with men and women in the general population, snowball sampling was used where recruited individuals could recommend others who fit the criteria. For FGDs with the general population, half took place in urban areas of each district and the other half in rural areas to ensure the viewpoints of both rural and urban men and women were considered. Individuals with comorbidities were recruited by district hospital staff who ran monthly clinics for people living with comorbidities. Health workers were recruited through focal points from the MOH at the district level, and community gatekeepers were recruited through district health offices and HSAs. There were no refusals.

### Data Collection

Data collectors were trained by the project’s research manager and the study coordinator with support from MOH representatives. Guides were piloted during 1 half-day near the training site, after which the group debriefed and made minor revisions to improve wording and flow. Data collection activities were facilitated by 4 teams, 2 for FGDs and 2 for IDIs. Each FGD team was composed of a facilitator, a notetaker, and a transcriber, and each IDI team likewise included a facilitator and a notetaker who also transcribed. One FGD team and 1 IDI team collected data in Machinga and Karonga districts. The other FGD team and IDI team collected data in Thyolo and Ntchisi districts. All data collection activities were audio-recorded and transcribed verbatim with simultaneous translation into English.

### Data Analysis

Two members of the Breakthrough ACTION team conducted a thematic analysis of transcripts.^11^ After an initial data familiarization phase that included reading and memoing (a process of reflective notetaking^12^) 7 transcripts (16%) to develop a codebook, analysts coded all transcripts in Atlas.ti 9. Codes were both deductive (from the interview guides) and inductive (developed through the data familiarization stage and coding), resulting in 43 codes grouped into 6 overall topics: (1) COVID-19 risk perception, (2) prevention, testing, and experiences of COVID-19, (3) factors influencing vaccination decisions, (4) health workers’/gatekeepers’ roles and relationships, (5) COVID-19 vaccine promotion, and (6) COVID-19 vaccine delivery. Each analyst reviewed a selection (4) of the other analyst’s transcripts and provided feedback, discussing any questions to ensure coding consistency. Analysts then extracted data by code and reviewed the output, identifying themes and illustrative quotations through a collaborative process of memoing, discussions, and report writing. Themes were sentences that summarized an insight about the topic. For example, “unvaccinated groups were afraid of the side effects/power of the vaccine and effect of numerous doses” was a theme related to factors influencing vaccination decisions. A subset of the themes related to efforts to integrate COVID-19 vaccination into other routine or emergency vaccine programs. For this purpose, the analysis focused on the following 3 questions.

1. How worried are people about COVID-19 relative to other health threats like cholera, and why?
2. How confident are people about COVID-19 vaccines relative to other vaccinations with which they might be bundled for promotion and delivery? What factors influence vaccine confidence?
3. What aspects of the specialized COVID-19 vaccine delivery helped or hurt vaccine uptake that could inform a successful integrated approach?

### Ethical Approval

The activity and secondary analyses of the data were reviewed and approved by the Johns Hopkins School of Public Health Institutional Review Board.

## RESULTS

The team conducted a total of 27 FGDs: 12 with individuals vaccinated against COVID-19, 10 with individuals unvaccinated against COVID-19, 2 with social workers, and 3 with people who had comorbidities. The team also conducted a total of 16 IDIs: 8 with health workers at COVID-19 vaccination sites and 8 with community gatekeepers (chiefs, village heads, representatives). The total sample included 224 adult participants, 47% of whom were females (Table).

**TABLE.** Study Method and Participants of the Qualitative Assessment to Explore COVID-19 Vaccine Perceptions in Malawi

	**No. of Participants**		
	**Male**	**Female**	**Total**
**Focus group discussions (N=27)**			
Unvaccinated (n=10)	37	38	75
Vaccinated (n=12)	47	48	95
Social workers (n=2)	10	6	16
People with comorbidities (n=3)	10	12	22
Subtotal	104	104	208
**In-depth interviews (N=16)**			
Health workers (n=8)	6	2	8
Gatekeepers (n=8)	7	1	8
Subtotal	13	3	16
TOTAL	117	107	224

In the following sections, we summarize the participants’ viewpoints with respect to their risk perception for COVID-19 as opposed to cholera or other health threats, factors that influenced vaccine confidence, and reflections on the way COVID-19 vaccines were promoted and delivered.

### How Worried Are People About COVID-19 Relative to Other Health Threats Like Cholera, and Why?

When asked to list the health issues that concern them most, participants in the FGDs listed a variety of diseases as well as poverty, hygiene, and availability of medicines at health facilities. At the time of data collection, cholera had not yet spread to many districts, but participants still felt cholera was a threat. Health workers likewise described cholera as both deadly and difficult to contain. Malaria, HIV/AIDS, hypertension, and diabetes also topped the list and were perceived to affect communities indiscriminately. COVID-19 was occasionally mentioned as a top concern but typically was omitted or mentioned after other, more pressing issues. When prompted, some members of the general population described COVID-19 as a very threatening disease. The lack of treatment or inaccessibility of treatment was a characteristic that increased the perceived severity of top health issues, particularly COVID-19 and cholera.

Participants occasionally mentioned COVID-19 as a top health concern but typically omitted it or mentioned it after other, more pressing issues.

*Cholera is very dangerous because when someone has cholera, they don’t last a lot of hours before they die if they don’t receive treatment.* —Vaccinated FGD, female, Machinga

Health workers in the study tended to feel COVID-19 was a serious issue, both due to their own occupational exposure and the fact that, at the time, it was “a disease without medication,” as a health worker in Thyolo noted.

Vaccinated groups emphasized the novelty of COVID-19 and described a sense of confusion not knowing where the virus came from or if people had it. A cough, once considered to be a small concern, over the course of the pandemic had become a threatening experience.

*The COVID disease...is the most confusing of all the illnesses that come in our bodies. Because if we talk of suffocating, it is there, whether it is coughing, COVID is there; whether fever, it is there; and all these makes one vulnerable to this disease. Once one shows signs of these illnesses, that’s where we have the COVID confusion, we are unable to know the illness we have; is it COVID, maybe or is it just a cough?* —Vaccinated FGD, male, Machinga

*Coughs started a long time ago, but COVID is what has confused our intelligence.* —Vaccinated FGD, male, Karonga

Hearing of people with a serious case of COVID-19 appeared to increase risk perception on top of the novelty.

COVID-19 risk perception tended to be lower among unvaccinated groups, with many describing COVID-19 as equivalent to a cold. Two participants agreed that “we simplify it because it’s just coughing and running nose.” A female in Machinga in the unvaccinated FGD added, “When we get sick, we just go to the grocery and buy medicine/drugs and get well.” People who felt cholera or other diarrheal diseases were less concerning emphasized the belief that there was only a true risk during the rainy season and that people who were informed could prevent the illness through hygiene practices.

According to participants in both FGDs and IDIs, risk perception ebbed and flowed over the course of the pandemic and often was not consistent enough to prompt some people to get vaccinated. Certain members of unvaccinated groups simply did not see the importance of getting the COVID-19 vaccine.

*They tell us that you must go and get vaccinated, but we refuse because we don’t know the benefits of getting vaccinated*. —Unvaccinated FGD, male, Karonga

The perceived irrelevance of the vaccines mostly reflected low perceived susceptibility or, less commonly, perceived severity.

*Here most of us believe we will not contract COVID, that’s why we are not interested in getting a vaccine.* —Unvaccinated FGD, male, Ntchisi

In contrast, participants in FGDs described the fear of cholera as a driver of vaccine uptake.

*They organize [public address] systems and megaphones and sensitize people that there is a cholera vaccine activity taking place; and people flock to receive the vaccine – in high numbers. Because with cholera deaths increase during a season such as we are approaching, and people are usually afraid. Actually, there is no one left behind, all people go. If you come here, you would find that this whole place is filled up. Give me the vaccine, give me the vaccine, give me the vaccine, they say … all because they are afraid of dying from cholera.* —Unvaccinated FGD, male, Machinga

Compared to COVID-19, there was higher fear of cholera with high perceived susceptibility and severity. Supported by widespread awareness-raising activities when the cholera vaccine was available, these fears appeared to motivate people to show up for OCV.

### How Confident Are People About COVID-19 Vaccines Relative to Other Vaccinations With Which They Might Be Bundled for Promotion and Delivery? What Factors Influence Vaccine Confidence?

Vaccine confidence encompasses people’s beliefs about the benefits and safety of vaccines as well as their trust in the medical system and motivations of those who deliver vaccinations.^10^^,^^13^ Participants in the study generally understood the benefit of COVID-19 vaccines as preventing severe illness and death, even if a person was infected.

*When vaccinated, the sickness of vaccinated person is different from the sickness of unvaccinated one, so the vaccine is just good*. —Health worker, female, IDI, Karonga

Health workers tended to be most well informed about the function of vaccines, but gatekeepers and members of the general population were also able to describe broadly what vaccines do. Vaccinated and unvaccinated groups did not substantially differ in their understanding of why the COVID-19 vaccines are important.

*I heard that it’s for protection, that when you are infected, the disease will not have a big impact on your body—that there is going to be a difference in the seriousness of the disease between the vaccinated and unvaccinated. —*Unvaccinated FGD, female, Machinga

Although the understanding of the function of COVID-19 vaccines was similar, the balance of advantages and risks with respect to the vaccine versus COVID-19 disease weighed differently. For people who had already gotten at least 1 dose of the COVID-19 vaccine, they perceived the vaccines as both beneficial and safe. Vaccinated participants (and health workers) tended to put the COVID-19 vaccines in the same category as other vaccines and to not see substantial differences. People expressed feeling comfortable with the COVID-19 vaccines because they had received many vaccines as a child.

People who had already gotten at least 1 dose of the COVID-19 vaccine perceived the vaccines to be both beneficial and safe.

*Because I am vaccinated, I am similar to children who get vaccinated with Vitamin A, also polio, I am protected similarly.* —Gatekeeper, male, IDI, Karonga

*COVID-19 vaccine is a vaccine that is like any other vaccine that is introduced in Malawi. We are just pleading with all our brothers and sisters to go and get vaccinated. It is the same as the vaccine we give to children at the under 5 clinic, to men and other diseases, there is nothing new.* —Vaccinated FGD, male, Karonga

When vaccinated participants were asked why people received other vaccines but not those for COVID-19, they speculated that it had to do with the rumors and misinformation, such as the idea that the vaccines were sent to depopulate African countries. Some vaccinated participants confirmed this perspective, saying that COVID-19 was the only vaccine where people had heard a lot of rumors.

*You have problems in bed, you get cold, and with that it’s only this vaccine [COVID-19] that has lots of this.* —Unvaccinated FGD, male, Karonga

For unvaccinated groups, the vaccines often were not mentioned as a way to prevent COVID-19 until prompted. Individuals in unvaccinated FGDs tended to mention severe side effects of the vaccine without mentioning the benefits. The number of doses was a concern for people.

*I had some concerns about doses of the vaccine, like AstraZeneca and JJ, I was trying to think that if I get 2 or 3 doses what will happen in my body?*—Unvaccinated FGD, male, Thyolo

In the risk/benefit calculation and within a low-perceived-risk environment, some members of the general population preferred to face COVID-19 if infected rather than get a vaccine.

*Is the vaccine used to reduce or treat the disease? They said it reduces the impact of the disease. If it was to treat at least everyone would have gotten the jab. In addition, maybe I will not be infected therefore there is no need to receive the vaccine.* —Unvaccinated FGD, male, Ntchisi

Gambling on avoiding infection was an appealing course of action in scenarios when risk perception was low and the risks of vaccination felt high. In fact, some unvaccinated individuals in FGDs described serious turmoil about the decision to get vaccinated or not as they walked through their risk/benefit analysis. In contrast, people with comorbidities generally were more concerned about the impact of COVID-19 disease than potential side effects from the vaccine.

In differentiating between cholera, polio, measles, and other vaccines, the main difference FGD participants mentioned was whether the vaccines were intended for children or adults. Vaccinated participants also mentioned the benefit of COVID-19 vaccines not leaving a scar. However, participants rated the COVID-19 vaccines unfavorably against other vaccines, including cholera, tetanus toxoid (often given during pregnancy), and polio. Other vaccines were perceived to have lighter side effects, be easier to deliver (by mouth rather than injection), and be both necessary and more established (not newly developed).

*[Tetanus toxoid vaccine] is a must and no one can run away from it, while COVID-19 vaccines can be escaped.* —Vaccinated FGD, male, Thyolo

*[Tetanus toxoid vaccine] is administered to a pregnant mother because of pregnancy, while COVID-19 vaccine is administered to people who are not sick, which makes people differentiate… for the pregnant women when they go for the antenatal care clinic, they go under detailed health education where they understand a lot of things, while for COVID-19 vaccines there is no such thing.* —Vaccinated FGD, male, Thyolo

Participants in Thyolo tended to feel they were less knowledgeable about the COVID-19 vaccine.

*We are well informed about these other vaccines [more] than COVID-19 vaccine. —*Unvaccinated FGD, male, Thyolo

There were even unfavorable comparisons among health workers, with a health worker asking why there were multiple brands of COVID-19 vaccines.

*We have always had vaccines for example for polio, but it was only 1 and the world has only that 1 polio vaccine and not multiple. For COVID there is Johnson and Johnson, Pfizer and many more.* —Health worker, male, IDI, Machinga

Others expressed doubts about the efficacy of the vaccine, particularly in light of changing recommendations around the number of doses.

*The concern is that people’s trust in the vaccine is waning away, for example when one is told to come for a booster dose, people were asking questions like what exactly do you want? You said when a person has done 2 doses the person is protected, now you are saying a third dose - it means your vaccine is fake.* —Health worker, female, IDI, Karonga

Health workers wanted more support in knowing how to explain why there are different brands and doses.

*I would want to learn about the types, we should we have these options, how are they different and why couldn’t they make just 1… I would want to know to also be able to answer what the people are inquiring.* —Health worker, male, IDI, Machinga

Despite that perception, participants in FGDs and gatekeepers described trusting health workers to help answer their questions and overcome concerns with respect to cholera and other health issues.

*Our trusted information source and the ones who do not get tired are these, our health workers here… The main reason we trust them most was in terms of hygiene, the issue in lake side, cholera. They are the ones who work on that a lot.* —Gatekeeper, male, IDI, Machinga

Health workers set an example for people who feared the newly authorized COVID-19 vaccine.

*[Health workers] move around the communities showing us vaccination cards saying, “Look we have received the vaccination so if it’s dying, we will die.” When we saw they were not dying, we then felt like we should do the same.* —Gatekeeper, male, IDI, Thyolo

Although, in general, participants trusted health workers, some raised questions about the motives and COVID-19 vaccine confidence of health workers.

*Health care workers were advising people not to vaccinate, when walking round they could shout about the vaccine to motivate people to vaccinate, but when reaching people’s home, that’s when they were talking in their low voice to say that people should not vaccinate*. —Comorbidities FGD, male, Thyolo

Health workers—while generally feeling that the community trusted them—also described incidents of mistreatment or stigmatization related to COVID-19 or cholera.

*If we have a cholera funeral, we wrap the body in a plastic paper then straight for burial, similar case with COVID-19 funeral … [we] were close to being beaten in the communities; people said these people just kill people.* —Health worker, female, IDI, Karonga

### What Aspects of the Specialized COVID-19 Vaccine Delivery Helped or Hurt Vaccine Uptake That Could Inform a Successful Integrated Approach?

Two topics emerged regarding COVID-19 vaccine-specific strategies that were likely detrimental to uptake. First, members of the general population in FGDs expressed that vaccine promoters, health leaders, and national leaders emphasized that the COVID-19 vaccines were optional and a personal choice each person should make. Although not necessarily convinced that the COVID-19 vaccines should have been mandatory, participants felt this framing undermined the expected normative nature of vaccines, which consequently harmed vaccine confidence. Participants had the impression that health workers or leaders did not think the vaccine was important because otherwise, they would not have mentioned it as being a choice or right but rather as something everyone should do to protect the community, which was standard for other vaccines.

Participants felt that framing the vaccine as optional or a personal choice undermined the expected normative nature of vaccines, which consequently harmed vaccine confidence.

*The first to say it was the president, that this is everyone’s right, no one should blame the government for forcing them*. —Vaccinated FGD, male, Karonga

*Currently health care workers are saying it is supposed to be voluntary, administered not by force. So, people don’t take it seriously and the words of saying “It’s not by force” has discouraged a lot of people to vaccinate.* —Vaccinated FGD, male, Thyolo

The emphasis on choice over duty was confusing for communities.

*A lot of people were struggling to understand … health workers seemed like you were helping to demotivate people by telling them it was not by force. By telling them it was not by force, they said it was their choice not to get it. … The message came in a wrong way, telling people that it is their right. They should have just been told to go and get the vaccine because the disease would be there. Instead, they used to say it is everyone’s right, even to not get it, it’s your right.* —Vaccinated FGD, male, Machinga

Community members pointed out that requiring vaccination may not have been at the level of a national mandate or a policy but could have come from local leaders as a local “command.” However, the level of trust in both local leaders and the government was a key factor in how people framed the concept of choice with respect to the vaccines. In certain cases, gatekeepers indicated that pushing for vaccines caused people to think that the leaders were being paid or were trying to “sell” their constituents.

A second issue FGD participants raised was the COVID-19 vaccination card. Having a separate card rather than including COVID-19 vaccination in existing vaccine records or health passports/books related to the Essential Programme on Immunization concerned people.

*I had this other question which still bothers me up to this day, when we suffer from malaria and other diseases which require an injection, they are all indicated in the health passport/book. Why is it that with a COVID-19 injection one is provided with a separate card? Many people are saying a lot about this card. Some say that once you have received the card that means you have accepted to join 666, up until now I don’t have an answer to that question.* —Unvaccinated FGD, female, Karonga

The vaccination card was linked to the issue of mandates and individual choice. For some, the COVID-19 vaccine cards were helpful to enter public spaces where vaccination was required. A participant described only changing his mind about getting vaccinated if the card was required.

*I will not get vaccine … unless if there will be restrictions on doing things like accessing the hospital, shops, traveling, I will need a card. At that time I will get the vaccine.* —Unvaccinated FGD, male, Ntchisi

In contrast, certain participants found the COVID-19 vaccine card to be problematic, particularly with the rumors circulating about the mark of the beast or the end times.

*When you get COVID-19 vaccine, you are given a card while other vaccines you don’t, so this surprises people; to say why only the COVID-19 vaccine is given a separate card, while the rest of the vaccines are written in the health passport? We are scared that it’s connected to Satan.* —Unvaccinated FGD, female, Ntchisi

Even though Malawi also has a dedicated vaccination card for cholera, participants did not bring that up as a concern for OCV.

Although there were differing opinions across these 2 themes, there was an overall sense that treating COVID-19 vaccination like any other vaccine without a separate card would have been useful while also deemphasizing the “personal choice” narrative around COVID-19 vaccination. Concretely, people felt it would have been beneficial to offer COVID-19 vaccines in the course of routine health services and without distinguishing it with special strategies. A participant described an easy experience getting vaccinated for COVID.

*Because I left home and came here to receive treatment for another illness, then I found them administering COVID-19 vaccine, so I was like, let me just get vaccinated. —*Vaccinated FGD, female, Machinga

A participant suggested following the plan that polio vaccination teams use.

*I think the polio vaccine, when being administered, they were moving door by door, unlike the COVID-19 vaccine. COVID-19 vaccine is done in villages and where they find a lot of people they administer there.* —Vaccinated FGD, male, Karonga

Participants with comorbidities suggested working through existing clinics and support groups that they already attend to offer COVID-19 vaccines and information. Social workers and gatekeepers in IDIs suggested that door-to-door and community-based delivery would not be in opposition to integration efforts.

*Health workers go around the community and vaccinated. They also integrate with other programs like under-5 clinic.* —Social worker FGD, male, Ntchisi

Although they were in favor of door-to-door delivery rather than fixed-site delivery to increase convenience and address barriers related to distance, health workers emphasized the burden on vaccinators to cover a large geographic area and make it to hard-to-reach areas.

## DISCUSSION

Our study uses qualitative data collected during the cholera outbreak that started in March 2022, which opened the opportunity for participants to speak about OCV and other adult vaccines as they discussed COVID-19 vaccination. The results contribute to the existing literature on COVID-19 vaccine intentions and uptake,^14^^,^^15^ general vaccination efforts, and vaccine hesitancy^16^ by providing insights about participants’ thought processes and resulting perceptions, fears, concerns, and reactions to a new vaccine for a disease that was not perceived as serious compared to cholera and that suffered from abundant misinformation and rumors.^17^

COVID-19 took the world by surprise, and its sometimes asymptomatic nature complicated its perceived prevalence and severity among communities around the world.^18^ Compared to cholera, which was reemerging in an explosive way and was well known for its severity, COVID-19 was not perceived as threatening, particularly among unvaccinated participants as the pandemic progressed into its third year. In the Malawi context, with low population density in large geographic areas where most people reside, distance may have prevented the spread, thus contributing to the low perceived risk.

Rumors and conspiracy theories that flooded social media about the alleged dangers of the COVID-19 vaccines combined with its elective nature created doubts and vaccine hesitancy, as vaccine fears outweighed the low perceived risk for the disease. Consistent with other research, our findings show that there were multiple reasons for vaccine hesitancy that could be case and content specific.^16^ Although there are no data that quantify the level of COVID-19 vaccine hesitancy in Malawi, it has been suggested that among Malawian adults, issues of vaccine safety, effectiveness, and side effects were prevalent.^19^^,^^20^ These concerns may have affected general vaccine coverage in Malawi because parents worried that their children could be given the COVID-19 vaccine during routine immunization without their knowledge.^21^ Our study findings contribute key insights into some of the reasons behind these concerns.

Our findings show that there were multiple reasons for vaccine hesitancy and could be case and content specific.

Compared to well-established vaccines that have been promoted as necessary for the public good and delivered using outreach and door-to-door approaches,^17^ the COVID-19 vaccine being a personal choice and health facility-based left people puzzled and then concerned. These doubts were exacerbated when a separate vaccination card was created for this single vaccine rather than combining it with OCV vaccination cards, which could have leveraged people’s openness to OCV, or incorporating COVID-19 vaccination into the standard vaccination records in individuals’ health passports/books. Within this doubting environment, the dosing of the different vaccine brands added to people’s worries that too many doses meant the vaccine did not work as intended or was fake or that the vaccines would be too strong for their bodies. The unavoidable shifts in messaging as different brands and boosters were authorized were hard for people to understand.

In emergency situations, people need a trusted authority to direct them on what to do. Consistent with other COVID-19 vaccine research,^15^^,^^17^^,^^21^ the study participants valued and relied on their health workers. As it became clear that COVID-19 vaccination coverage was lagging due to supply and delivery issues, including logistics that made COVID-19 vaccine delivery more challenging than other vaccines and the proliferation of misinformation, promotional strategies shifted from a fixed-side modality to a door-to-door approach.^17^ Consistent with findings from our study, this community-focused approach improved uptake. Yet, some health workers were unable to answer questions about brands and doses or unable to allay people’s fears and concerns. Community members in our study described health workers quietly expressing negativity and distrust about the vaccines, consistent with other research on both COVID-19 and cholera vaccines, suggesting that health workers sometimes avoid promoting vaccination to clients.^22^^,^^23^ As the responsibility for vaccine promotion and delivery shifted to HSAs, who were trusted people in the community, organizations working in RCCE saw vaccine hesitation reduced, and vaccination rates started to increase.^17^ There are several lessons from the COVID-19 vaccine roll-out, and actions have been suggested to reduce the gap between willingness and actual uptake.^24^ For future emergencies, the results of our study imply that people will be more responsive to public health directives if these are consistent with standards of practice known to people in those contexts, are clarified when they deviate from such standards, are consistently reinforced by policymakers and influencers, and have mechanisms in place to expeditiously clarify misinformation and to keep health providers well informed on the nuances of emerging information.

### Limitations

There are a few limitations in this article. The analysis is based on a sample collected from only 4 districts in Malawi using qualitative methodology; therefore, findings do not necessarily apply to other settings. However, based on how consistent some of our findings are with other COVID-19 vaccine studies, we think that the insights from these specific settings contribute key information about how individuals conceptualize and make decisions about adult vaccination, which can inform strategies to integrate COVID-19 promotion and delivery with routine health services in similar settings. Participants may have answered questions in ways that made them “look good” to others in the group or the facilitator, as well as echoed the beliefs of others. This limitation was mitigated by encouraging participants that their honest opinions would help the country’s response to health threats. The consistency of findings across study sites indicates people offered honest opinions on the issues explored.

## CONCLUSION

As Malawi and other countries move toward integration of COVID-19 vaccination into routine health services or other emergency services, this study offers insights into challenges and opportunities that must be employed. The twindemic of cholera and COVID-19 compounded the suffering of communities already under strain in multiple ways, and the opportunity existed to bundle COVID-19 vaccine promotion within the cholera response. Integration of COVID-19 and cholera mobilization campaigns was both necessary and beneficial. Our study suggests the COVID-19 vaccine would benefit from being repositioned as a regular vaccine like others that people already receive, offered routinely to the general population, and more intensely promoted among the most vulnerable, as with influenza or pneumococcal vaccines. Beyond the cholera response, our findings indicate that simply offering COVID-19 vaccinations in the course of other services like antenatal care, door-to-door Essential Programme on Immunization campaigns, or hospital visits for other health issues could be enough to prompt vaccine uptake. Globally, health authorities can cascade training and messaging strategies that avoid emphasizing personal choice or highlighting COVID-19 as a separate type of threat but rather frame COVID-19 vaccination as similar to other routine vaccinations and a necessary measure for the public good.

## Supplementary Material

GHSP-D-23-00146-supplement1.pdf

GHSP-D-23-00146-supplement3.pdf

GHSP-D-23-00146-supplement2.pdf

## Notes

**Peer Reviewed**

**First published online:** December 1, 2023.
