# Supplementary material for: Perceptions of the COVID-19 Vaccine and Other Adult Vaccinations in Malawi: A Qualitative Assessment
Source: Glob Health Sci Pract. 2024 Feb 20;12(Suppl 1):e2300146. doi: 10.9745/GHSP-D-23-00146 (PMC10948124; doi:10.9745/GHSP-D-23-00146)
Supplement: GHSP-D-23-00146-supplement1.pdf [file GHSP-D-23-00146-supplement1.pdf]

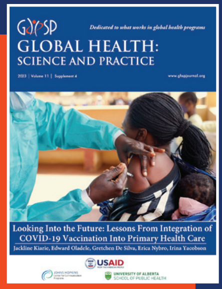

Perceptions of the COVID-19 Vaccine and Other Adult Vaccinations in Malawi: A Qualitative Assessment: Article Summary

Natalie Tibbels, Rominie Kaseghe, Alvin Blessings Chisambi, Vitima Ndovi, Alfred Mang'ando, Maria Elena Figueroa

What is this article about? In 2022, about 15% of Malawians were fully vaccinated for COVID-19, with coverage in some districts as low as 1%. This study explored perceptions that adults in Malawi had about the COVID-19 vaccine compared to other adult vaccines.

What were the results? Focus group discussions and interviews with both vaccinated and unvaccinated people in the general population, as well as health workers and community leaders, explored 3 questions:

1. How worried are people about COVID-19 relative to other health threats like cholera, and why?

Some adults feared cholera and other health issues more than COVID-19, which may have contributed to low uptake of the COVID-19 vaccine. Among those who were not vaccinated, they felt the risk of getting infected with COVID-19 was low.

"We simplify it [COVID-19], because it's just coughing and running nose."

"Cholera is "very dangerous because when someone has cholera, they don't last a lot of hours before they die if they don't receive treatment."

- Focus group participant

2. How confident are people about COVID-19 vaccines relative to other vaccinations with which they might be bundled for promotion and delivery? What factors influence vaccine confidence?

Some participants understood that the COVID-19 vaccine would prevent severe illness but still expressed concern about risks. Some people who were not vaccinated were concerned about severe side effects of the vaccine and often referenced rumors or misinformation.

Insights from the Authors

Breakthrough ACTION/Malawi was implemented by the Johns Hopkins Center for Communication Programs from 2020-2023 and was funded by USAID.

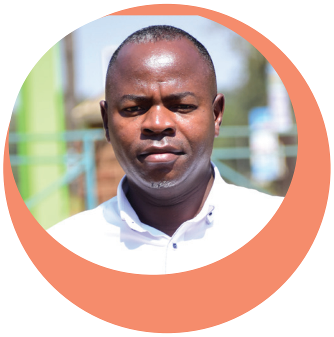

"Our study suggests the COVID-19 vaccine should be treated like other vaccines that people already receive, while making sure that community leaders and health workers have the information they need to deal with people's concerns."

- Rominie Kaseghe, MEL Officer, Breakthrough ACTION/Malawi

Read the original article published in Global Health Science and Practice for free at:
https://doi.org/10.9745/GHSP-D-23-00146

Trust in the COVID-19 vaccine was also affected by the multiple vaccine brands, changing guidance, and the need for additional boosters.

"We have always had vaccines for example for polio, but it was only 1 and the world has only that 1 polio vaccine and not multiple. For COVID there is Johnson and Johnson, Pfizer and many more."
—Health worker, Machinga

3. What aspects of the specialized COVID-19 vaccine delivery helped or hurt vaccine uptake that could inform a successful integrated approach?

Some participants also expressed distrust of the different strategies used to deliver COVID-19 vaccines, such as the need to have a separate vaccination card. The emphasis from leaders that the vaccine was optional, and a personal choice created confusion.

"Currently health care workers are saying it is supposed to be voluntary, administered not by force. So, people don't take it seriously and the words of saying "It's not by force" has discouraged a lot of people to vaccinate." – Vaccinated focus group participant

Other well-established vaccines in Malawi had been promoted as necessary for the public good and delivered through outreach and door-to-door approaches. However, rumors and misconceptions about the COVID-19 vaccine created fears, doubts, and hesitancy to get the vaccine. This, combined with the low perceived risk of getting COVID-19 and vaccine messaging that emphasized personal choice fueled more doubts and concerns.

What do these results mean?

- Vaccines may not be all be perceived in the same way. People may be more responsive to public health messages on new vaccines if the messaging is consistent with what they know about existing vaccines, is clear when it's different from what they know, is reinforced by policymakers and influencers, and can be clarified when new information is available
- The findings suggest the COVID-19 vaccine would benefit from being repositioned as a regular vaccine like others that people already receive, offered routinely to the general population, and more intensely promoted among the most vulnerable, as with influenza or pneumococcal vaccines.
- The study suggests that bundling the COVID-19 vaccine with other, more trusted vaccines or offering the vaccine while providing other health services could help increase uptake of the vaccine.

When and where was this study done?
At the time of the study in 2022, Malawi's health system was already strained by the COVID-19 pandemic when a cholera outbreak happened. Data were collected in four districts of Malawi (Ntchisi, Thyolo, Machinga, and Karonga) that had low vaccine coverage (between 1% and 11%) in August 2022.

USAID FROM THE AMERICAN PEOPLE

Knowledge SUCCESS

This summary brief is made possible by the support of the American People through the U.S. Agency for International Development under the Knowledge SUCCESS (Strengthening Use, Capacity, Collaboration, Exchange, Synthesis, and Sharing) Project Cooperative Agreement No. 7200AA19CA00001 with the Johns Hopkins University.
Knowledge SUCCESS is supported by USAID's Bureau for Global Health, Office of Population and Reproductive Health and led by the Johns Hopkins Center for Communication Programs (CCP) in partnership with Amref Health Africa, The Busara Center for Behavioral Economics (Busara), and FHI 360. The information provided in this summary brief are the sole responsibility of Knowledge SUCCESS and does not necessarily reflect the views of USAID, the U.S. Government, or the Johns Hopkins University.
