# Supplementary material for: Perceptions of the COVID-19 Vaccine and Other Adult Vaccinations in Malawi: A Qualitative Assessment
Source: Glob Health Sci Pract. 2024 Feb 20;12(Suppl 1):e2300146. doi: 10.9745/GHSP-D-23-00146 (PMC10948124; doi:10.9745/GHSP-D-23-00146)
Supplement: GHSP-D-23-00146-supplement3.pdf [file GHSP-D-23-00146-supplement3.pdf]

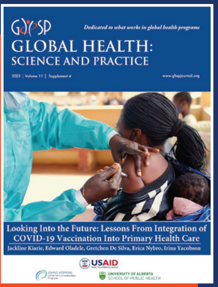

Perceções sobre a vacina contra a COVID-19 e outras vacinas para adultos no Malawi: uma avaliação qualitativa: resumo do artigo

Natalie Tibbels, Rominie Kaseghe, Alvin Blessings Chisambi, Vitima Ndovi, Alfred Mang'ando, Maria Elena Figueroa

De que trata este artigo? Em 2022, cerca de 15% dos malawianos foram totalmente vacinados contra a COVID-19, com uma cobertura em alguns distritos tão baixa como 1%. Este estudo explorou as perceções que os adultos do Malawi tinham sobre a vacina contra a COVID-19 em comparação com outras vacinas para adultos.

Quais foram os resultados? As discussões em grupos de foco e as entrevistas com pessoas vacinadas e não vacinadas da população em geral, bem como com profissionais de saúde e líderes comunitários, exploraram 3 questões.

1.Até que ponto as pessoas estão preocupadas com a COVID-19 em relação a outras ameaças para a saúde, como a cólera, e porquê?
Alguns adultos temiam mais a cólera e outros problemas de saúde do que a COVID-19, o que pode ter contribuído para a baixa adesão à vacina contra a COVID-19. As pessoas que não foram vacinadas consideraram que o risco de serem infetadas com a COVID-19 era baixo.

> *"Simplificamos [a COVID-19] porque é apenas tosse e corrimento nasal."*
>
> *A cólera é "muito perigosa porque, quando alguém tem cólera, não dura muitas horas antes de morrer se não receber tratamento."*
>
> *- Participante do grupo de discussão*

2.Qual é o grau de confiança das pessoas nas vacinas contra a COVID-19 em relação a outras vacinas com as quais podem ser agrupadas para promoção e distribuição? Que fatores influenciam a confiança nas vacinas?
Alguns participantes compreenderam que a vacina contra a COVID-19

Perspetivas dos autores

O projeto Breakthrough ACTION/Malawi foi implementado pelo Johns Hopkins Center for Communication Programs de 2020 a 2023 e foi financiado pela USAID.

"O nosso estudo sugere que a vacina contra a COVID-19 beneficiaria se fosse reposicionada como uma vacina regular, como outras que as pessoas já recebem, oferecida rotineiramente à população em geral e promovida de forma mais intensa entre os mais vulneráveis."

**- Rominie Kaseghe, Director de RMEL
Breakthrough ACTION/Malawi**

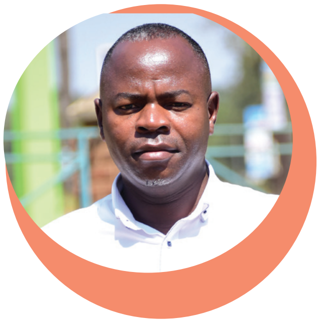

Leia o artigo original publicado na Global Health Science and Practice gratuitamente em:
https://doi.org/10.9745/GHSP-D-23-00146

preveniria doenças graves, mas ainda assim manifestaram preocupação com os riscos. Algumas pessoas que não foram vacinadas estavam preocupadas com os efeitos secundários graves da vacina e referiram frequentemente rumores ou informações incorretas. A confiança na vacina contra a COVID-19 foi também afetada pelas múltiplas marcas de vacinas, pela alteração das orientações e pela necessidade de reforços adicionais.

"Atualmente, os profissionais de saúde dizem que é suposto ser voluntária, e não administrada à força. Por isso, as pessoas não levam a sério e o facto de dizerem "Não é obrigatória" desencorajou muitas pessoas a vacinarem-se."

- Participante de um grupo de discussão sobre vacinação

"Sempre tivemos vacinas, por exemplo, para a poliomielite, mas era apenas uma e o mundo só tem essa vacina, e não várias. Para a COVID, há a Johnson and Johnson, a Pfizer e muitas outras."

- Profissional de saúde, IDI, Machinga

3. Que aspetos da administração especializada da vacina contra a COVID-19 ajudaram ou prejudicaram a adesão à vacina que poderiam informar uma abordagem integrada bem-sucedida?

Alguns participantes também manifestaram desconfiança em relação às diferentes estratégias utilizadas para administrar as vacinas contra a COVID-19, como a necessidade de ter um cartão de vacinação separado. A ênfase dada pelos líderes ao facto de a vacina ser opcional e uma escolha pessoal criou confusão.

Outras vacinas bem estabelecidas no Malawi tinham sido promovidas como necessárias para o bem público e distribuídas através de abordagens de proximidade e porta-a-porta. No entanto, os rumores e equívocos sobre a vacina contra a COVID-19 criaram receios, dúvidas e hesitação em tomar a vacina. Este facto, combinado com a baixa perceção do risco de contrair COVID-19 e as mensagens sobre vacinas que enfatizavam a escolha pessoal, alimentou mais dúvidas e preocupações.

O que significam estes resultados?

- As vacinas podem não ser vistas da mesma forma. As pessoas podem ser mais recetivas às mensagens de saúde pública sobre novas vacinas se estas forem coerentes com o que elas sabem sobre as vacinas existentes, se forem claras quando são diferentes do que sabem, se forem reforçadas pelos decisores políticos e pelos influenciadores e se puderem ser clarificadas quando estiverem disponíveis novas informações.

- Os resultados sugerem que a vacina contra a COVID-19 beneficiaria se fosse reposicionada como uma vacina regular, como outras que as pessoas já recebem, oferecida por rotina à população em geral e promovida mais intensamente entre os mais vulneráveis, como acontece com as vacinas contra a gripe ou pneumocócicas.

- O estudo sugere que a agregação da vacina contra a COVID-19 a outras vacinas mais fiáveis, ou a oferta da vacina ao mesmo tempo que se prestam outros serviços de saúde poderia ajudar a aumentar a adesão à vacina.

Quando e onde foi efetuado este estudo? Na altura do estudo, em 2022, o sistema de saúde do Malawi já estava sob pressão devido à pandemia de COVID-19, quando ocorreu um surto de cólera. Os dados foram recolhidos em quatro distritos do Malawi (Ntchisi, Thyolo, Machinga e Karonga), que tinham uma baixa cobertura vacinal (entre 1% e 11%) em agosto de 2022.

USAID FROM THE AMERICAN PEOPLE

Knowledge SUCCESS

Este guia resumido é possível graças ao apoio do povo americano através da U.S. Agency for International Development ao abrigo do Acordo de Cooperação do Projeto Knowledge SUCCESS (Strengthening Use, Capacity, Collaboration, Exchange, Synthesis, and Sharing) n.º 7200AA19CA00001 com a Universidade Johns Hopkins. O Knowledge SUCCESS é apoiado pelo Gabinete de Saúde Global, da População e Saúde Reprodutiva da USAID e dirigido pelo Johns Hopkins Center for Communication Programs (CCP) em parceria com a Amref Health Africa, The Busara Center for Behavioral Economics (Busara) e a FHI 360. As informações fornecidas neste guia resumido são da exclusiva responsabilidade da Knowledge SUCCESS e não refletem necessariamente as opiniões da USAID, do Governo dos EUA ou da Universidade Johns Hopkins.
