# Supplementary material for: Perceptions of the COVID-19 Vaccine and Other Adult Vaccinations in Malawi: A Qualitative Assessment
Source: Glob Health Sci Pract. 2024 Feb 20;12(Suppl 1):e2300146. doi: 10.9745/GHSP-D-23-00146 (PMC10948124; doi:10.9745/GHSP-D-23-00146)
Supplement: GHSP-D-23-00146-supplement2.pdf [file GHSP-D-23-00146-supplement2.pdf]

Leçons tirées de l’intégration de la vaccination contre la COVID-19 dans les soins de santé primaires | Série de résumés d’articles

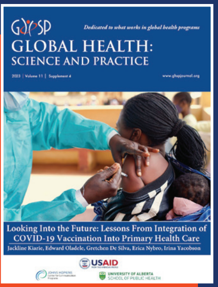

Perceptions du vaccin contre la COVID-19 et d’autres vaccins pour adultes au Malawi : Une évaluation qualitative : Résumé de l’article

Natalie Tibbels, Rominie Kaseghe, Alvin Blessings Chisambi, Vitima Ndovi, Alfred Mang’ando, Maria Elena Figueroa

De quoi parle cet article ? En 2022, environ 15 % des Malawiens étaient complètement vaccinés contre la COVID-19, la couverture dans certains districts n’atteignant que 1 %. Cette étude a exploré les perceptions que les adultes du Malawi avaient sur le vaccin contre la COVID-19 par rapport à d’autres vaccins pour adultes.

Quels étaient les résultats ? Des discussions de groupe et des entretiens avec des personnes vaccinées et non vaccinées de la population générale, ainsi qu’avec des agents de santé et des responsables communautaires, ont permis de répondre à trois questions.

1. Dans quelle mesure la COVID-19 inquiète-t-elle par rapport à d’autres menaces sanitaires comme le choléra, et pourquoi ?

Certains adultes craignaient davantage le choléra et d’autres problèmes de santé que la COVID-19, ce qui a pu contribuer à la faible utilisation du vaccin en question.

« Nous la prenons à la légère [COVID-19], car il s’agit simplement d’une toux et d’un écoulement nasal. »

Le choléra est « très dangereux parce que lorsqu’une personne est atteinte par cette maladie, elle meurt peu de temps après si elle n’est pas soignée. »

— Participant au groupe de discussion

Les personnes non vaccinées estimaient que le risque de contracter le virus était faible.

2.Dans quelle mesure la population a-t-elle confiance dans les vaccins contre la COVID-19 par rapport à d’autres vaccins avec lesquels ils pourraient être regroupés pour la promotion et l’administration ? Quels sont les facteurs qui influencent la confiance dans les vaccins ?

Certains participants ont compris que le vaccin contre la COVID-19 permettrait

Points de vue des auteurs

Breakthrough ACTION/Malawi a été mis en œuvre par Johns Hopkins Center for Communication Programs de 2020 à 2023 et financé par l’USAID.

« Notre étude suggère que le vaccin contre la COVID-19 soit traité comme les autres vaccins déjà administrés, tout en veillant à ce que les responsables communautaires et les agents de santé disposent des informations nécessaires pour répondre aux inquiétudes de la population.»

- Rominie Kaseghe, responsable de RMEL Breakthrough ACTION/Malawi

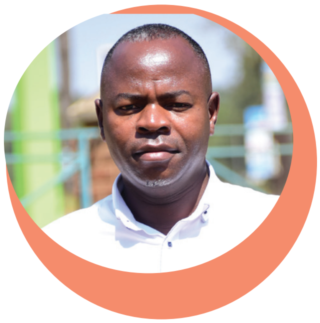

Lisez gratuitement l’article original publié dans Global Health Science and Practice à l’adresse suivante : https://doi.org/10.9745/GHSP-D-23-00146

de prévenir une maladie grave, mais ont tout de même exprimé des inquiétudes quant aux risques. Certaines personnes non vaccinées s'inquiétaient des effets secondaires graves du vaccin et faisaient souvent référence à des rumeurs ou à des informations non fondées. La confiance dans le vaccin contre la COVID-19 a également été affectée par la multiplicité des marques de vaccins, l'évolution des directives et la nécessité de rappels supplémentaires.

« Nous avons toujours eu des vaccins, par exemple pour la polio, mais il ne s'agissait que d'un seul vaccin contre la polio et dans le monde entier, il n'y a qu'un seul vaccin contre la polio et non plusieurs. En ce qui concerne la COVID, il y a Johnson and Johnson, Pfizer et bien d'autres. »

–Agent de santé, IDI, Machinga.

3. Quels sont les aspects de l'administration spécialisée du vaccin contre la COVID-19 qui ont favorisé ou entravé l'absorption du vaccin et qui pourraient servir de base à une approche intégrée réussie ?

Certains participants ont également exprimé leur méfiance à l'égard des différentes stratégies utilisées pour administrer les vaccins contre la COVID-19, comme la nécessité de disposer d'une carte de vaccination distincte. L'accent mis par les dirigeants sur le fait que le vaccin était facultatif et qu'il s'agissait d'un choix personnel a semé la confusion.

«Actuellement, les professionnels de la santé affirment qu'il s'agit d'une mesure volontaire, qui ne doit pas être administrée de force. Les gens ne prennent donc pas cela au sérieux et le fait de dire « Ce n'est pas obligatoire a découragé beaucoup de gens de se faire vacciner. »

— Participant au groupe de discussion

D'autres vaccins bien établis au Malawi ont été présentés comme nécessaires au bien public et distribués par le biais d'approches de proximité et de porte-à-porte. Cependant, les rumeurs et les idées fausses concernant le vaccin contre la COVID-19 ont suscité des craintes, des doutes et une certaine hésitation à se faire vacciner. Cette situation, combinée à la faible perception du risque de contracter la COVID-19 et aux messages sur les vaccins qui mettaient l'accent sur le choix personnel, a alimenté les doutes et les inquiétudes.

Que signifient ces résultats ?

- Les vaccins ne sont pas tous perçus de la même manière. La population peut être plus réceptive aux messages de santé publique sur les nouveaux vaccins si ces messages sont cohérents avec les connaissances qu'elle a des vaccins existants, s'ils sont clairs lorsqu'ils diffèrent de ce qu'elle sait, s'ils sont renforcés par les décideurs politiques et les personnes influentes et s'ils peuvent être clarifiés lorsque de nouvelles informations sont disponibles.

- Les résultats suggèrent que le vaccin contre la COVID-19 gagnerait à être repositionné comme un vaccin ordinaire, comme d'autres vaccins déjà administrés, proposé systématiquement à la population générale et promu plus intensément auprès des personnes les plus vulnérables, comme c'est le cas pour les vaccins antigrippaux ou antipneumococciques.
- L'étude suggère que l'association du vaccin contre la COVID-19 à d'autres vaccins plus fiables ou l'offre du vaccin en même temps que d'autres services de santé pourraient contribuer à augmenter l'utilisation du vaccin.

Quand et où cette étude a-t-elle été réalisée ? Au moment de l'étude en 2022, le système de santé du Malawi était déjà mis à rude épreuve par la pandémie de la COVID-19 lorsqu'une épidémie de choléra s'est déclarée. Les données ont été recueillies dans quatre districts du Malawi (Ntchisi, Thyolo, Machinga et Karonga) qui présentaient une faible couverture vaccinale (entre 1 % et 11 %) en août 2022.

USAID FROM THE AMERICAN PEOPLE · Knowledge SUCCESS

Ce guide a été réalisé grâce au soutien du peuple américain par l'intermédiaire de l'Agence américaine pour le développement international dans le cadre du projet Knowledge SUCCESS (Strengthening Use, Capacity, Collaboration, Exchange, Synthesis, and Sharing, accord de coopération n° 7200AA19CA00001 avec Johns Hopkins University. Knowledge SUCCESS est soutenu par le Bureau de la santé mondiale de l'USAID, le Bureau de la population et de la santé reproductive, et dirigé par le John Hopkins Centre for Communications Programs (CCP) en partenariat avec Amref Health Africa, le Centre d'économie comportementale de Busara et « FHI 360 ». Les informations fournies dans cette ressource relèvent de la seule responsabilité de Knowledge SUCCESS et ne reflètent pas nécessairement les opinions de l'USAID, du gouvernement américain ou de Johns Hopkins University.
